# *Rhizoma coptidis* as a Potential Treatment Agent for Type 2 Diabetes Mellitus and the Underlying Mechanisms: A Review

**DOI:** 10.3389/fphar.2019.00805

**Published:** 2019-07-22

**Authors:** Qian Ran, Jin Wang, Lin Wang, Hai-rong Zeng, Xiang-bo Yang, Qin-wan Huang

**Affiliations:** ^1^College of Pharmacy, Chengdu University of Traditional Chinese Medicine, Chengdu, China; ^2^Ya’an Xun Kang Pharmaceutical Co., Ltd, Ya’an, China

**Keywords:** Rhizoma coptidis, Huang Lian, type 2 diabetes mellitus, complications, ethnopharmacology

## Abstract

Diabetes mellitus, especially type 2 diabetes mellitus (T2DM), has become a significant public health burden. *Rhizoma coptidis* (RC), known as *Huang Lian*, is widely used for treating diabetes in China. The bioactive compounds of RC, especially alkaloids, have the potential to suppress T2DM-induced lesions, including diabetic vascular dysfunction, diabetic heart disease, diabetic hyperlipidemia, diabetic nephropathy, diabetic encephalopathy, diabetic osteopathy, diabetic enteropathy, and diabetic retinopathy. This review summarizes the effects of RC and its bioactive compounds on T2DM and T2DM complications. Less research has been conducted on non-alkaloid fractions of RC, which may exert synergistic action with alkaloids. Moreover, we summarized the pharmacokinetic properties and structure-activity relationships of RC on T2DM with reference to extant literature and showed clearly that RC has potential therapeutic effect on T2DM.

## Introduction

Diabetes is an incurable metabolic disorder which affects more than 135 million people worldwide (Zhu and Zhang, 2016). It has been estimated that nearly 95% of diabetics worldwide are type 2 diabetes mellitus (T2DM) (Jhong et al., 2015; Thomas and Philipson, 2015). The characteristics of T2DM are mainly insulin resistance and insufficient insulin secretion, which result in high levels of blood glucose (DeFronzo, 2004). Long-term hyperglycemia results in inflammation, oxidative stress, and other pathological changes, which eventually lead to multiple organ injury, including diabetic nephropathy, diabetic neuropathy, and diabetic cardiomyopathy (Forbes and Cooper, 2013; Konig et al., 2013; Rios et al., 2015; Labazi and Trask, 2017). At present, the therapeutic strategies used for diabetes are mainly insulin injection and administration of chemical hypoglycemic drugs. However, these treatments are associated with adverse side effects, such as gastrointestinal problems and hypoglycemia (Teng and Chen, 2017). Many traditional Chinese medicines have been used in treating T2DM. It is of interest that these traditional medicines have acceptable preventive and therapeutic effects on diabetic complications through their multiple constituents and multiple routes of action (Chen et al., 2018).

**Figure 1:**
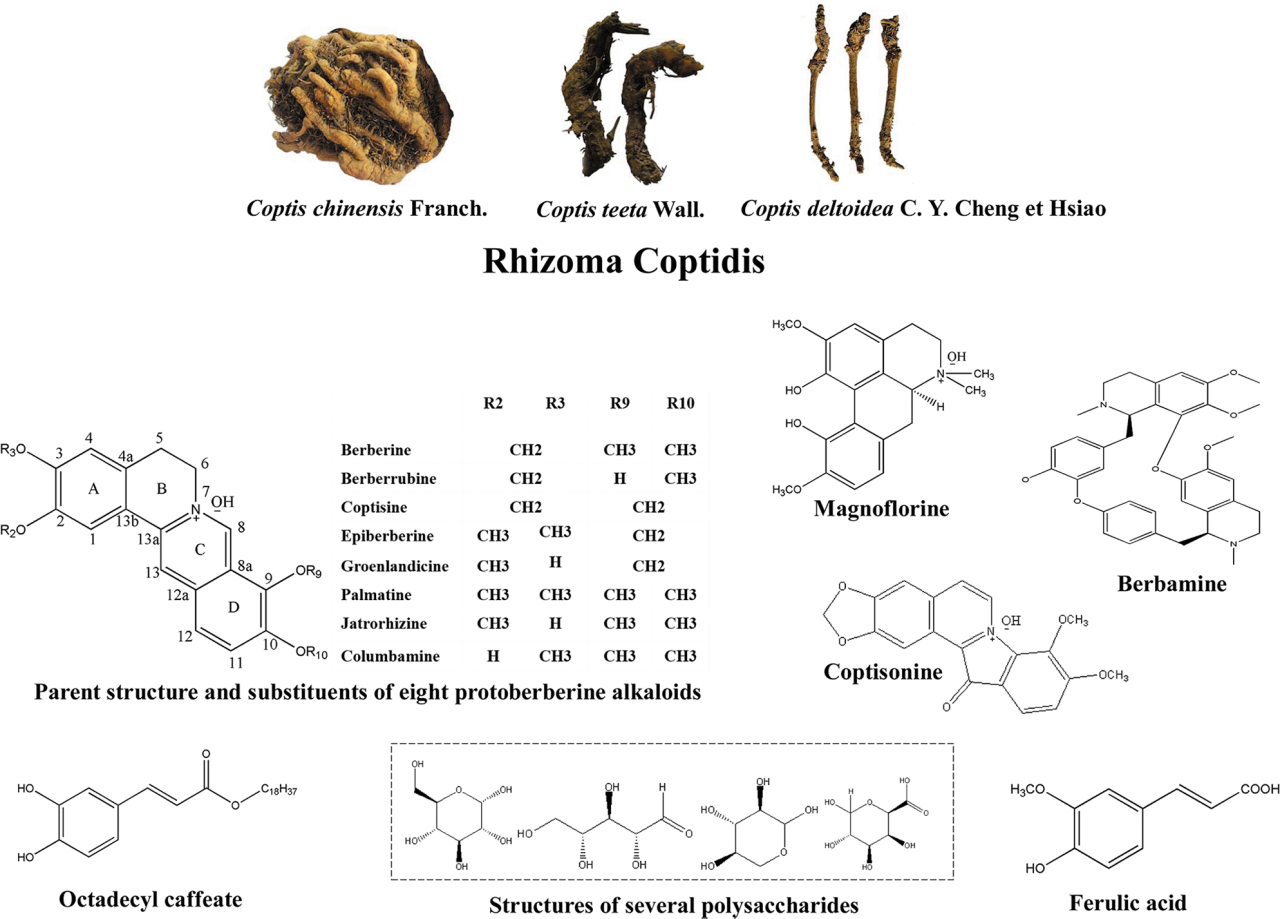
Appearance of *Coptis chinensis* Franch., *Coptis deltoidea* C. Y. Cheng et Hsiao and *Coptis teeta* Wall, and the structures of bioactive anti-T2DM components of RC.

*Rhizoma coptidis* (RC) is the dried rhizome of medicinal plants from the family *Ranunculaceae: Coptis chinensis* Franch., *Coptis deltoidea* C. Y. Cheng et Hsiao, and *Coptis teeta* Wall (**Figure 1**) (Tan et al., 2016). RC and its processed products *Jiu-huanglian* have been used against diabetes in China for more than 1,400 years (Zhou et al., 2008; Wang et al., 2014). Many famous formulas containing RC, such as *San-Huang-Xie-Xin-Tang* (Wu et al., 2016), and *Jiao-Tai-Wan* (Chen et al., 2013a), have also been shown to exert therapeutic effects on T2DM. More than 100 chemical constituents have been identified in RC. These include alkaloids, simple phenylpropanoids, flavonoids, and other compounds (Wang et al., 2019). Researches of RC in T2DM have mainly focused on berberine, coptisine, palmatine, epiberberine, jatrorrhizine, and magnoflorine. Other alkaloids, including berberrubine, columbamine, and berbamine, also have been shown to possess anti-diabetic potential. Non-alkaloidal constituents of RC, such as polysaccharides and polyphenols, also have potential therapeutic effects on T2DM and may promote the effects of alkaloids (Chen et al., 2012; Jiang et al., 2013).

**Figure 2:**
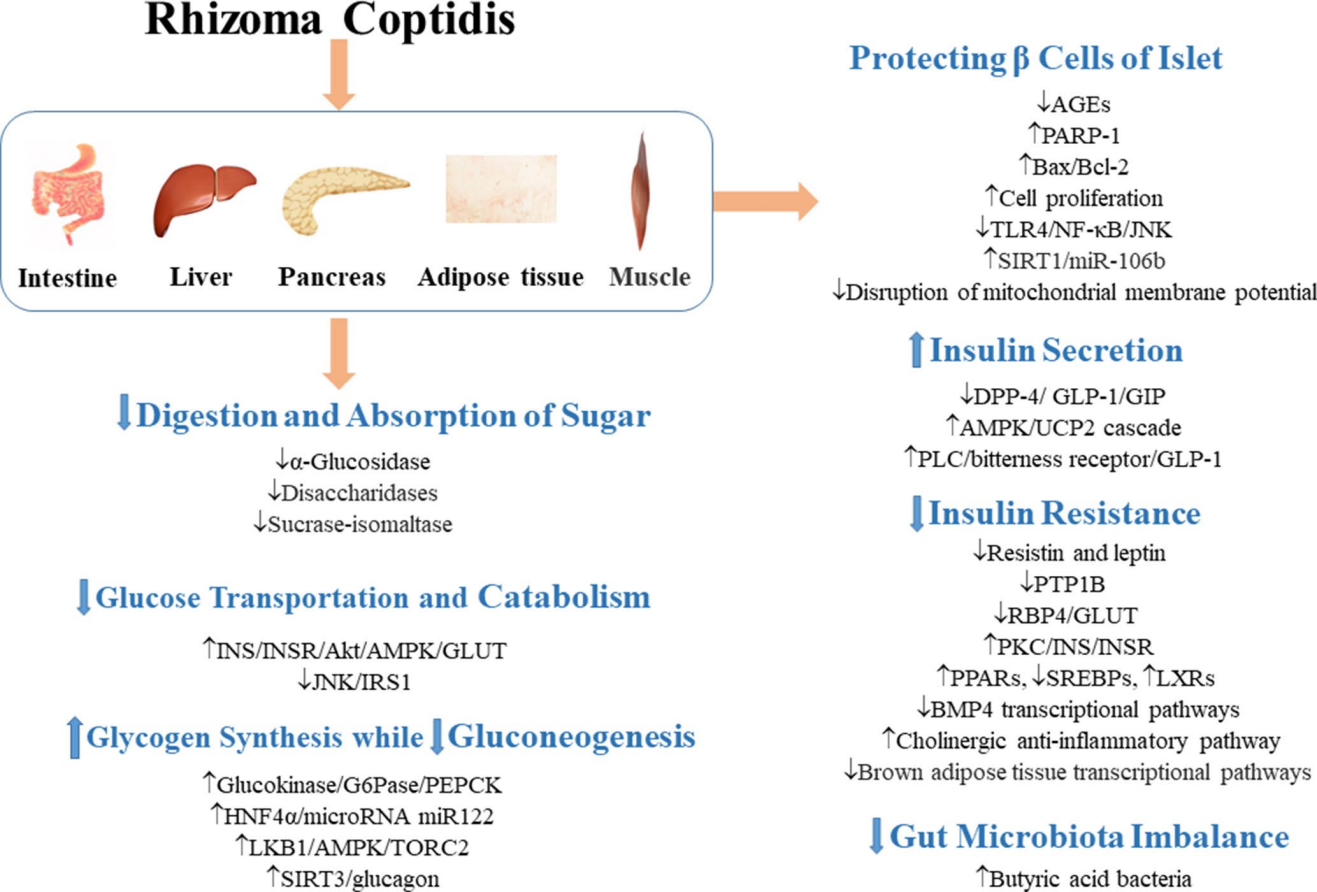
Mechanisms involved in the regulation of glycometabolism in T2DM by RC. ↓ indicates inhibition/reduction, while ↑ indicates increase/promotion. The up or down direction of arrows reflects the promotion or suppression of the first signal in the signaling pathway.

**Figure 3:**
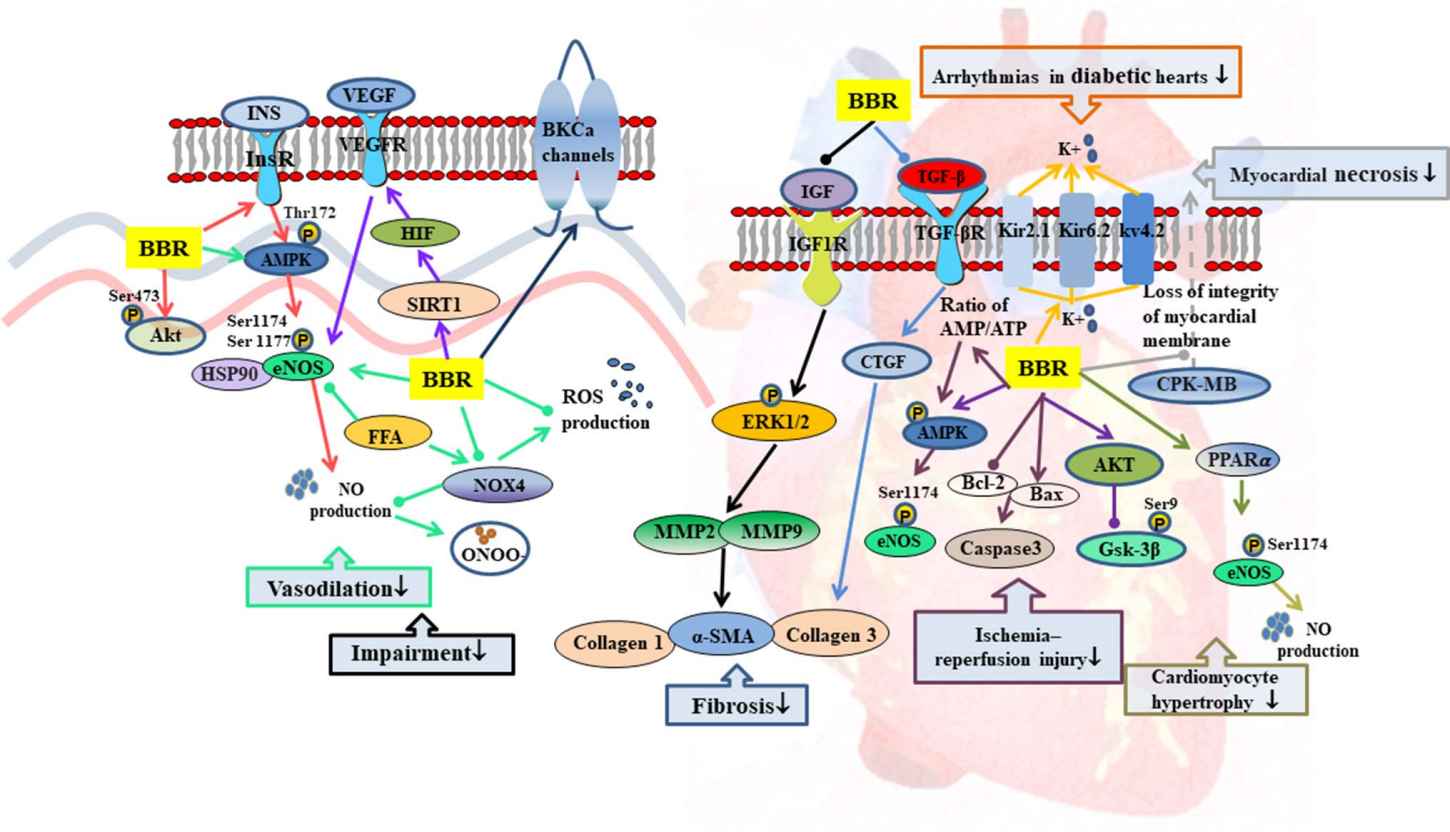
Mechanisms of RC on diabetic vascular dysfunction and diabetic heart disease. —• with different colors indicate inhibition/reduction while → with different colors indicate increase/promotion. Arrows of different colors depict different references, and when the results of mechanism research overlap, just randomly select one of the colors. BBR represents berberine.

**Figure 4:**
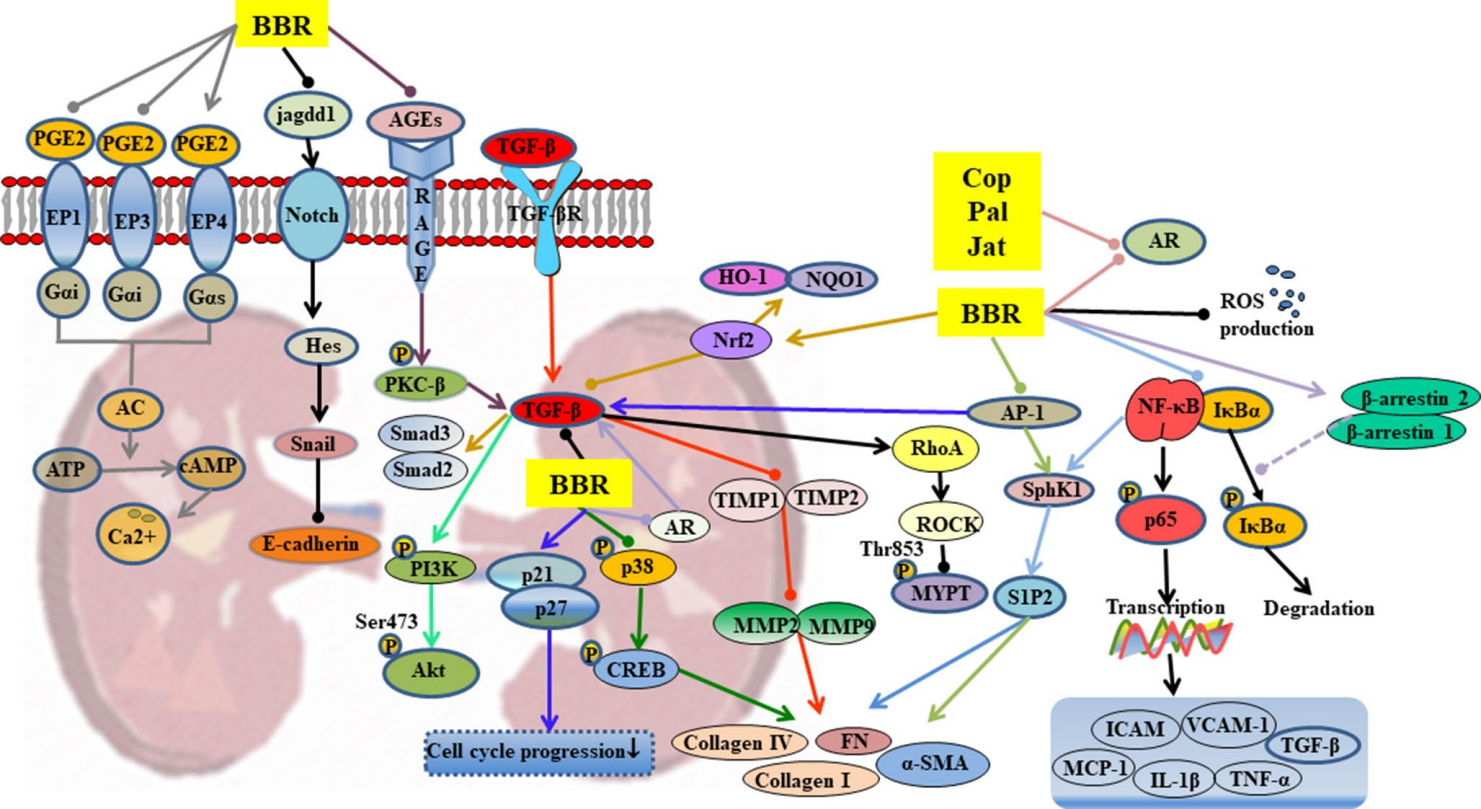
Mechanisms of RC on diabetic nephropathy. —• with different colors indicate inhibition/reduction while → with different colors indicate increase/promotion. Arrows of different colors depict different references, and when the results of mechanism research overlap, just randomly select one of the colors. BBR, Cop, and Jat represent berberine, coptisine, and jatrorrhizine.

**Figure 5:**
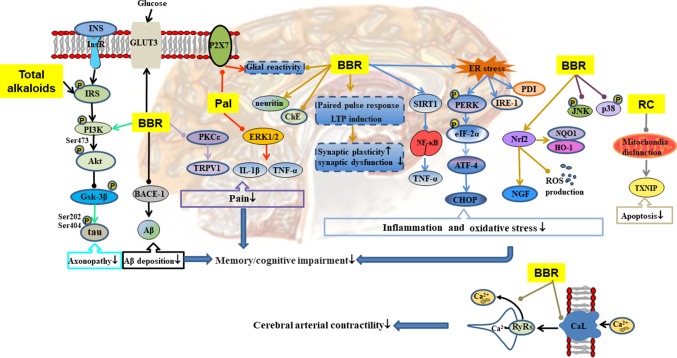
Mechanisms of RC on diabetic encephalopathy. —• with different colors indicate inhibition/reduction while → with different colors indicate increase/promotion. Arrows of different colors depict different references, and when the results of mechanism research overlap, just randomly select one of the colors. BBR and Pal represent berberine and palmatine.

**Figure 6:**
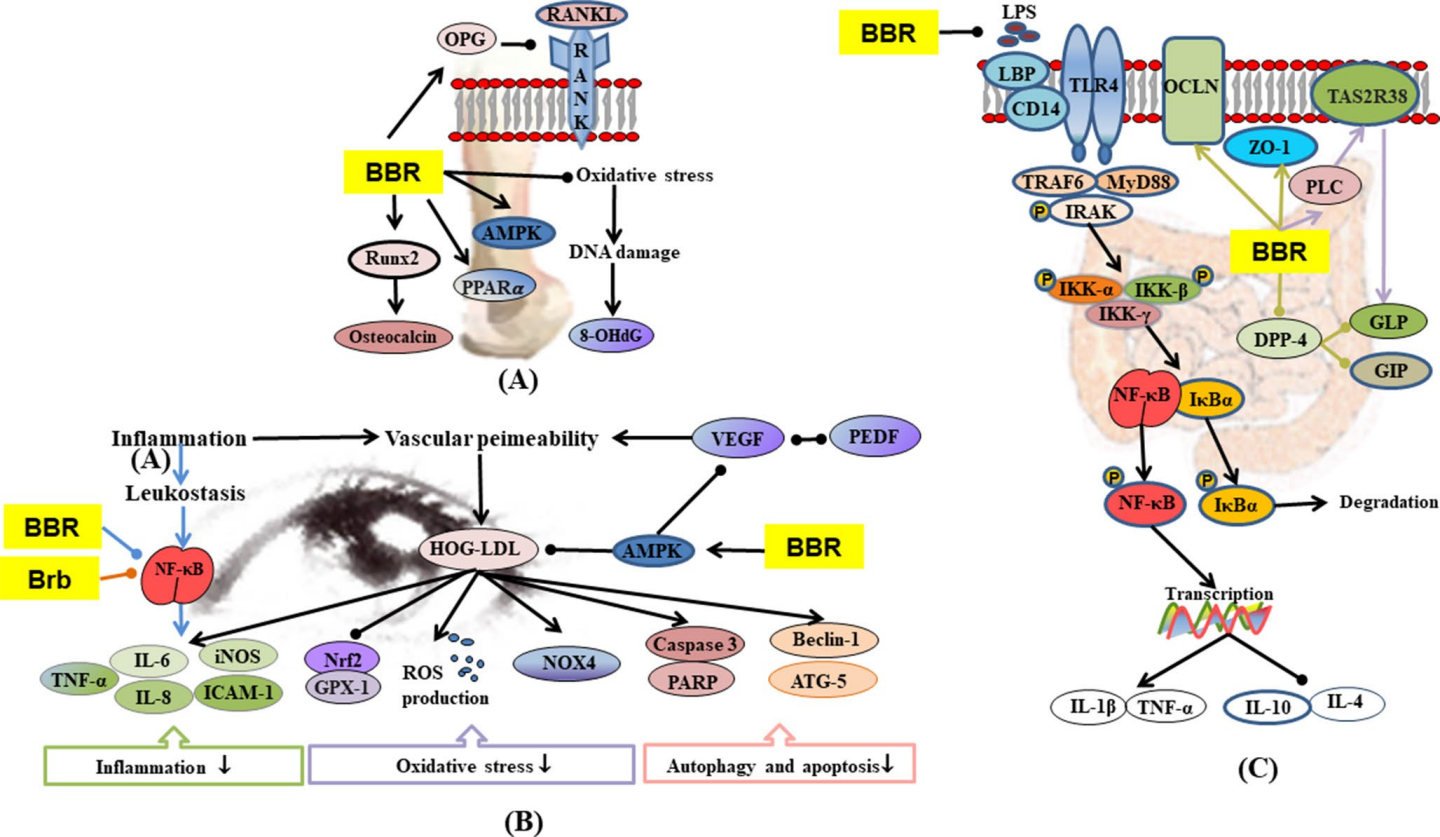
**(A)** Mechanisms of RC on diabetic osteoporosis; **(B)** mechanisms of RC on diabetic retinopathy; **(C)** mechanisms of RC on diabetic enteropathy. —• with different colors indicate inhibition/reduction while → with different colors indicate increase/promotion. Arrows of different colors depict different references, and when the results of mechanism research overlap, just randomly select one of the colors. BBR and Brb represent berberine and berberrubine.

Given the increasing populations of T2DM patients, the potential benefits in exploration of new drugs for T2DM have attracted many researchers to carry out in-depth studies on the effect of RC on T2DM and T2DM complications. As shown in **Figures 2**–**6** and **Table 1**, RC regulates various signaling pathways and regulates the expressions of proteins and genes in many tissues and organs. Thus, it has good preventive and therapeutic effects on the complications of T2DM. However, the underlying mechanisms are still largely unknown. More molecular studies in the future are expected to elucidate the definite mechanism of its actions, especially the synergism between non-alkaloids and alkaloids. Large-scale clinical researches are necessary for the evaluation of its safe clinical dosage, interaction with other drugs, and specific indications in T2DM.

**Table 1:** Effects of RC and its bioactive compounds on T2DM complications.

Diabetic complications	Drug/ extract	Model	Dose and treat time	Described effects	Anti-diabetic mechanism	Ref
**Diabetic vascular dysfunction**	BBR	Wistar rats, STZ + high-fat diet (HFD)	100 mg/kg/day, 8w	FBG and TG levels↓; endothelium-dependent vasorelaxation impaired in aorta↓	eNOS mRNA and protein↑ and NOX4 protein expressions↓ in aortas	(Wang et al., 2009a)
	BBR	SD rats, STZ + HFD; human artery endothelial cells, high glucose+palmitate	200 mg/kg/day, 4 weeks; 2.5–10 µmol/L	Insulin-induced vasodilatation↑; cell viability and autophagy↑	Phosphorylation of the insulin receptor and its downstream AMPK, Akt, eNOS↑; down-regulating insulin receptors attenuated BBR-induced p-AMPK.	(Geng et al., 2016)
	BBR	Human umbilical vein endothelial cells, 30 mM glucose	25 µM, 60 min	Endothelium-dependent vasodilatations↑	Interaction between eNOS and HSP90↑; NO production↑; NO release by berberine is eNOS and AMPK-dependent; phosphorylation of the a-catalytic subunit of AMPK at its activation site (Thr172)↑; cGMP levels in the isolated rat aorta↑	(Wang et al., 2009b)
	BBR	Human umbilical vein endothelial cells, palmitate	1.25, 2.5, and 5 µmol/L, 24 h		NO levels↑; production of ROS↓; NOX4 protein expression↓; expression of eNOS↑; protein expressions of AMPK and p-AMPK↑	(Zhang et al., 2013)
	BBR	SD rat, STZ + HFD; cerebral vascular smooth muscle cells, 20 mM D-glucose	100 and 200 mg/kg/day, 8 weeks; 10 µM, acute extracellular application	Blood glucose↓; systolic and diastolic blood pressure↓; relaxation in the presence of 20 mM D-glucose↑	Large-conductance Ca^2+^-activated K^+^ channel and expression of β1-subunit at protein or mRNA levels↑	(Ma et al., 2017)
	BBR	Microvascular endothelial cells, hypoxic/high‐glucose	30 µM, 24 h	Proliferation and migration↑	DPP-4 expression↓; expressions of VEGF, eNOS, HIF-1α, and SIRT1↑	(Mi et al., 2018)
**Diabetic heart disease**	BBR	SD rat, STZ + HFD	200 mg/kg/day, 4 weeks	Cardiac fibrosis and dysfunction↓	IGF-1R expression in cardiac fibroblasts↓; MMP-2/MMP-9, α-SMA, and collagen I expressions in diabetic hearts↓	(Li et al., 2018a)
	BBR	SD rat, STZ	100 and 150 mg/kg/day, 12 weeks	FBG↓, left ventricular systolic pressure and left ventricular end diastolic pressure absolute value↓; heart mass index and degree of cardiac fibrosis↓	Expressions of TGF-β1, CTGF, collagen 1 and collagen 3↓	(Lu et al., 2016)
	BBR	Rat primary cardiomyocyte, high glucose and insulin	1, 3, and 10 µM, 48 h	Cardiomyocyte hypertrophy↓	Expressions of PPARα, eNOS, and NO↑; activation of PPARα could directly modulate the expression of eNOS.	(Wang et al., 2013b)
	BBR	Wistar rats, STZ + HFD; H9c2 cells, palmitate	100 mg/kg/day, 16 weeks; 10 µM, 48 h	Systolic and diastolic dysfunction↓; fasting blood insulin↑, FBG, TC, and TG levels↓; cardiac collagen deposition↓	Cardiac TGF-β expression↓; pAMPK/AMPK, pAKT/AKT, and pGSK3β/GSK3β↑; α-myosin heavy chain (α-MHC) expression↑; β-MHC expression↓	(Chang et al., 2015)
	BBR	SD rat, STZ + ischemia/reperfusion	10 mg/kg/day (i.p.), 6 weeks	Blood glucose↓; body weight↑; recovered the hemodynamic parameters	Protein expression of Kir6.2 subunits in the diabetic hearts↑	(Kaya et al., 2018)
	BBR	Wistar rats, STZ + HFD + ischemia/reperfusion	100 mg/kg/day, 7 days	Ischemia-reperfusion injury infarct size and arrhythmia↓; serum TG, TC, and MDA levels↓	AMPK activity in nonischemic areas↑; AKT↑, GSK3β↓, ratio of AMP/ATP↑, and adenosine diphosphate to ATP↑in nonischemic areas	(Chang et al., 2016)
	BBR	Wistar rats, isoproterenol+STZ	100 mg/kg/day, 30 days	Blood glucose↓; body weight↑; liver, kidney, and islets damage↓; TC, TG, and LD↓; HDL↑	HbA1c (hemoglobin a1c) and isoenzyme of creatine phosphokinase (CPK-MB) levels↓; creatinine (Cr) and ALT levels ↓	(Suman et al., 2016)
	BBR	SD rats, STZ+ ischemia	100 mg/kg/day, 7 days	Arrhythmia↓; QTc interval↓; diminished I(to) and I(Ca) current densities↑	Decreased Ito in ischemic hearts was associated with the expression of Kv4.2	(Wang et al., 2012)
	BBR	SD rat, STZ + HFD+ coronary artery occlusion	180 mg/kg/day, 14 days	Recovering resting membrane potential; I(K1) current and current density↑; electrophysiological disturbance↓,	Expression of I(Kir2.1)↑	(Wang et al., 2011)
	BBR	SD rats, STZ + HFD+ ischemia/reperfusion; neonatal rat	100, 200, and 400 mg/kg/day, 4 weeks	Cardiac systolic/diastolic function↑; myocardial apoptosis↓in diabetic rats; hypoxia/reoxygenation-induced myocardial apoptosis↓	Bcl-2/Bax ratio↑; caspase-3 expression↓; activation of PI3K-Akt↑; AMPK and eNOS phosphorylation↑	(Chen et al., 2014)
**Diabetic hyperlipidemia**	RC extract	SD rat, STZ + HFD	7.88 g/kg/day, 30 days	Blood lipid level↓; insulin resistance↓	The expressions of SREBP-1c and SCAP in liver↓	(Liu et al., 2018)
	Polysaccharides	ICR mice, STZ + HFD	25, 50, and 100 mg/kg/day, 28 days	FBG, TG, and TC↓;	SOD and CAT activities in pancreas↑; MDA content in pancreas↓	(Jiang et al., 2013)
	Polysaccharides	Wistar rats, STZ + HFD	20, 50, and 100 mg/kg/day, 28 days	FBG, TG, TC↓	GSH-Px, SOD, and CAT activities↑; GSH and MDA contents↓; JNK and phospho-IRS1 expression↓; expression of phospho-PI3Kp85 and GLUT-4↑	(Jiang et al., 2015a)
	Pal, Jat	KK-Ay mice; HepG2 cells	225 mg/kg/day, 40 days; 5 mg/ml, 24 h	Food and water intake↓; TC and TG↓; HDL-C↑		(Ma et al., 2016a)
	Jat	Obesity mice	20 and 100 mg/kg/day, 8 weeks	Liver-to-body weight ratio and pathological alterations of liver↓; serum aspartate aminotransferase (AST), alanine aminotransferase (ALT), TG, TC and LDL-C levels↓, glucose tolerance, and insulin sensitivity↑	Hepatic mRNA levels of SREBP-1c and FAS↓; PPAR-α and CPT1A mRNA expressions↑	(Yang et al., 2016a)
	Col	Syrian golden hamsters, high fat and cholesterol diet	23.35, 46.7, 70.05 mg/kg/day, 33 days	TC, TG, and LDL-C↓; HDL-C↑; conversion of cholesterol to bile acids↑	CYP7A1 activation↑; mRNA and protein expressions of FTF, HNF-4α↑	(Wang et al., 2016b)
	Brb	HepG2 cells	15 µm, 24 h	Intracellular cholesterol contents↓	Hepatic PCSK9↓ and LDLR↑ *via* the ERK1/2 signal pathway	(Cao et al., 2018b)
	BBR	KK-Ay mice	225 mg/kg/day, 40 days	Serum HDL-C↑		(Ma et al., 2016a)
	BBR	Golden hamster, HFD	50 and 100 mg/kg/day, 6 weeks	Plasma TC, TG, LDL-C, and FFA content↓; plasma MDA and ApoB level↓; SOD level↑	mRNA expression of skeletal muscle GLUT4↑; liver LDLR mRNA expression↓	(Liu et al., 2015)
	BBR	Wistar rats, STZ + HFD; 3T3-L1 cells;	150 and 300 mg/kg/day, 16w; 4 µM, 8 days	Lipid accumulation↓	PPARα/δ/γ, CDK9 and cyclin T1 mRNA and protein expressions in adipose tissue↑; lipoprotein lipase(LPL) activity↑; activating protein 2 (aP2), TNF-α, and FFA↓	(Zhou and Zhou, 2010)
**Diabetic nephropathy**	BBR	SD rat, STZ	100 and 200 mg/kg/day, 8 weeks	FBG↓; BUN, urinary total protein (UTP), and Cr↓; ECM and renal fibrosis↓	Ratio of MMP2/TIMP2 and MMP9/TIMP1↑; TGFβ1, FN, and collagen IV expressions↓	(Ni et al., 2015)
	BBR	SD rat, STZ; mesangial cells, high glucose	200 mg/kg/day, 12 weeks; 10, 30, and 90 µM, 24 h	Restored metabolic parameters and renal morphology; MDA level↓; SOD activity↑	RhoA-GTP and its substrate p-MYPT↓; levels of IκBα↑; NF-κBp65↓; intercellular adhesion molecule-1(ICAM-1)↓; TGF-β1 and FN overproduction↓; ROS generation↓	(Xie et al., 2013)
	BBR	SD rat, STZ; primary glomerular mesangial cells, high glucose	200 mg/kg/day, 12 weeks	Typical symptoms (more eating, drinking, urine, less body weight)↓; FBG, BUN, Cr, 24h albuminuria, and kidney weight/body weight ratio↓	mRNA and protein expressions of S1P2 receptor↓; S1P2 receptor mediated FN expression↓; SphK1 activity and S1P production↓; NF-κBp65 nuclear translocation↓; NF-κB specific inhibitor obviously decreased the expression of S1P2	(Huang et al., 2012)
	BBR	C57BL/6 mice, alloxan	300 mg/kg/day, 12 weeks	FBG, kidney/body weight ratio, BUN, serum creatinine and 24h albuminuria↓; renal hypertrophy↓	TGF-β1 synthesis, FN, and Col IV accumulation↓; SphK1 expression and S1P production↓	(Lan et al., 2010)
	BBR	Mesangial cells, high glucose	10, 30, and 90 µM, 48 h		Activity and expression of SphK1↓; expressions of α-SMA, FN, TGF-β1, and AP-1↓; SphK1 was regulated by AP-1	(Lan et al., 2012)
	BBR	SD rat, STZ + HFD; mesangial cells, high glucose	10, 50, and 200 mg/kg/day, 4 weeks; 30, 60, and 90 µM, 24 h	FBG and body weight↓; the majority of biochemical and renal function parameters and histopathological changes↓	Production of AGEs↓ induced levels of RAGE, P-PKC-β, and TGF-β1 in injured kidneys↓	(Qiu et al., 2017)
	BBR	Glomerular mesangial cells, high glucose; podocytes	50 and 100 µM, 24 h	Podocytes injury caused by exosomes derived from high-glucose-induced glomerular mesangial cells↓	Inhibiting transfer of TGFβ1 from the glomerular mesangial cells to the podocytes through TGFβ1-PI3K/AKT pathway	(Wang et al., 2018c)
	BBR	SD rat, STZ + HFD	100 and 200 mg/kg/day, 8 weeks	Histopathological changes↓; markers of kidney dysfunction↓	ICAM-1 and VCAM-1 levels in the kidneys↓; β-arrestin 1 and β-arrestin 2↑	(Tang et al., 2016)
	BBR	Wistar rats, STZ + HFD	25 mg/kg/day, 20 weeks	Blood glucose↓; lipid deposition within the diabetic kidney↓; urinary excretion of albumin, thickening of GBM and renal fibrosis↓;	Pro-inflammatory cytokines (IL-1β, TNF-α) and chemokine (MCP-1) ↓; Smad3 signaling (FN, collagen I, collagen IV) and NF-κB signaling↓	(Sun et al., 2015)
	BBR	Rat glomerular mesangial cells, high glucose	30,90 µM, 24 h		Phospho-p38MAPK and phospho-CREB levels↓; FN and collagen synthesis↓	(Liu et al., 2009)
	BBR	Mesangial cells, high glucose	10, 30, and 90 µM, 24 h	Mesangial cell area↓; high-glucose-induced cell cycle progression↓; proliferation and hypertrophy↓	Cells in G1-phase↑ and in S-phase↓; p21(Waf1)/(Cip1) and p27(Kip1)↑; expressions of TGF-β1 and FN↓; transcription activity of NF-κB and AP-1↓	(Lan et al., 2014)
	BBR	SD rat, STZ + HFD	100 and 200 mg/kg/day, 6–8 weeks	Urine creatinine (UTP/C), BUN, and Cr levels↓; pathological changes, thickening of GBM, and mesangial matrix accumulation↓; inflammatory cell infiltration↓	EP4, Gαs, and cAMP levels↑	(Yang et al., 2014b)
	BBR	SD rat, STZ + HFD	50, 100, and 200 mg/kg/day, 8 weeks	Restoring renal functional parameters; alterations in histological and ultrastructural changes↓ in the kidney tissues, glucose and lipid metabolism disorders↓	Levels of IL-6 and PGE2↓; total protein expressions of EP1 and EP3 of renal cortex↓; expressions of EP4 and cAMP↑	(Tang et al., 2014)
	BBR	Wistar rats, STZ	100 and 200 mg/kg/day, 8 weeks	FBG↓; glomerular mesangial cells proliferation↓	Gαs protein↑; Gαi protein↓; cAMP level↑; secretion of TGF-β, collagen IV ↓; FN and CTGF synthesis↓	(Tang et al., 2013)
	BBR	SD rat, STZ + HFD	100 and 200 mg/kg/day, 8 weeks	Restoring renal functional parameters, alterations in histological and ultrastructural changes↓ in the kidney tissues; glucose and lipid metabolism disorders↓	cAMP level↑; protein expressions of GRK2 and GRK3↓; protein expressions of GRK6, GRK4↑; no significant change of GRK5	(Wang et al., 2013a)
	BBR	Wistar rats, STZ	200 mg/kg/day, 12 weeks	Glomerular area, glomerular volume, FBG, BUN, serum creatinine, and urine protein for 24h↓; MDA content↓; activity of SOD↑	AR mRNA and protein in the kidney↓	(Liu et al., 2008b)
	BBR	SD rat, STZ + HFD; podocytes, high glucose	100 and 200 mg/kg/day, 8 weeks; 30 µM, 24 h	Renal injury↓; ratio of kidney weight to body weight, 24 h urinary protein, serum creatinine, and BUN↓; systemic and renal cortex inflammatory response↓	IL-1β, IL-6, and MCP-1 expression↓; protein level of TLR4 and phophorylation of IκBα and p65↓	(Zhu et al., 2018)
	BBR	Wistar rats, STZ	400 mg/kg/day, 12 weeks	Kidney injury↓; glomerular hypertrophy and mesangial matrix expansion↓	mRNA and protein expressions of TGF-β, α-SMA, vimentin, NF-κB↓	(Li and Zhang, 2017)
	BBR	KKAy mice; mouse renal tubular epithelial cells, high glucose	150 mg/kg/day, 16 weeks; 30 µM, 48 h	Blood glucose and 24h urinary protein levels↓; degradation of renal function↓; normalization of an index of renal interstitial fibrosis and kidney weight/body weight; high-glucose-induced epithelial-to-mesenchymal transition events↓	α-SMA↓; E-cadherin levels↑; protein and mRNA levels of jagged1, notch1, and hes1↓; snail protein and mRNA expressions↓	(Yang et al., 2017a)
	BBR	SD rat, STZ + HFD	150 mg/kg, 8 weeks	24 h urinary microalbumin (mg) and urinary N-acetyl-glucosaminidase↓; renal tubulointerstitial injury↓	Expressions of α-SMA, NF-κB, and MCP-1↓; E-cadherin levels↑	(Ma et al., 2016c)
	BBR	C57BL/6J mice, STZ; NRK 52E cells, high glucose	200 mg/kg/day, 12 weeks; 30 µM, 48 h	Levels of FBG, Cr, BUN↓; renal fibrosis↓; epithelial-to-mesenchymal transition (EMT)↓	Nrf2/HO-1/NQO1 pathway↑; phospho-Smad2/3 and collagen I↓; inhibiting TGF-β/Smad/EMT signaling activity in Nrf2-dependent manner	(Zhang et al., 2016)
	BBR	SD rats, STZ; mesangial cells, high glucose	100 mg/kg/day, 8 weeks	Levels of UTP/C, BUN, and Cr↓; histopathological alterations↓; proliferation of mesangial cells↓	Abnormal concentration of cytoplasmic Ca^2+^, level of PGE2, the high expressions of EP1 and Gαq↓	(Ni et al., 2016)
**Diabetic encephalopathy**	CR extract	SH-SY5Y human neuroblastoma cells, tert-butylhydroperoxide	100 mg/ml, 2 and 24 h	Cell viability↑	Mitochondrial membrane potential (MMP)↑; thioredoxin-interacting protein (TXNIP)↓	(Friedemann et al., 2014)
	Total alkaloids	SD rat, STZ + HFD	80, 120, and 180 mg/kg/day, 24 weeks	Levels of FBG, glycosylated hemoglobin and glycosylated serum protein, FFA, TG, and TC↓; Aβ deposition↓; neuronal damage and loss↓; cognitive deficits↓	The phosphorylation of IRS, PI3K, and Akt↑; overactivation of GSK3β↓; content of ApoA1↑ and ApoB↓	(Li et al., 2018c)
	Pal	SD rat, STZ + HFD	30 mg/kg/day (i.p.), 14 days	Hyperalgesia, allodynia, and depressive behaviors↓; activation of satellite glial cells after nervous injury stimulus↓	Expressions of TNF-α and IL-1β in the hippocampus↓; colocalization of GFAP and P2X7 receptors ↓; phosphorylation of ERK1/2↓	(Shen et al., 2018)
	BBR	Wistar rats, STZ + HFD	100 mg/kg/day, 24 weeks	Body weight and blood levels of glucose↓; glycated hemoglobin, TG, TC↓; improved memory and affected evoked potential by decreasing latency	mRNA and protein expression of p38 and JNK↓; neuritin mRNA and protein levels↑; no effect on ERK1/2 protein	(Zhou et al., 2016)
	BBR	Wistar rat, STZ	50 and 100 mg/kg/day, 8 weeks	Body weights↑; serum glucose↓; MDA and nitrite levels in hippocampal homogenates ↓; SOD levels↑; hyperglycemia↓; astrogliosis↓	GFAP in the brain↓	(Moghaddam et al., 2014)
	BBR	Wistar rat, STZ	100 mg/kg/day, 12 weeks	Synaptic plasticity↑	Hippocampal CA1 neuronal apoptosis↓; long-term potentiation (LTP) induction↑; paired pulse facilitation↓	(Kalalian-Moghaddam et al., 2013; Moghaddam et al., 2013)
	BBR	SD rat, STZ + HFD; primary hippocampal neurons, high glucose	100 and 200 mg/kg/day, 10 weeks; 0.05, 0.1 µM, 1 h	Hyperglycemia and insulin resistance↓; MDA levels↓; SOD levels↑; memory impairment↓	Restoring PI3K/Akt/GSK3β signaling pathway; tau hyperphosphorylation↓	(Wang et al., 2018b)
	BBR	Wistar rats, STZ + HFD	187.5 mg/kg/day, 8 weeks	Inflammation mediator release and insulin resistance in medial prefrontal cortex↓; glucose uptake in the brain and metabolism of glucose in neuron↑; reinforcement of the information↑; cognitive impairment↓	PI3K/Akt/mTOR↑; PKCη and PKCε and nuclear translocation of NF-κB in neuron↓; neuron-specific glucose transporter GLUT-3↑; amyloid precursor protein and BACE-1↓; production of oligomeric Aβ42↓	(Chen et al., 2017b)
	BBR	SD rat, STZ; imprinting control region mice, STZ	20 and 60 mg/kg/day, 2 weeks; 30 and 90 mg/kg/day, 2 weeks	Hyperglycemia-induced inflammatory reaction↓; heat hyperalgesia threshold↑	Expressions of PKCε, TRPV1, TNF-α↓; inhibiting TRPV1 activation by blocking the PKC pathway	(Zan et al., 2017)
	BBR	db/db mice	50 mg/kg/day, 10 weeks	Learning and memory ability↑; cognitive impairment↓; FBG, TG, TC, and LDL‐C↓; HDL‐C↑	Synapse- and nerve-related protein expressions (PSD95, SYN, and NGF)↑; expressions of inflammatory factors (TNF-α and NF-κB) and ER stress-associated proteins (PERK, IRE-1α, eIF-2α, PDI, and CHOP) in the hippocampus↓; expression of SIRT1↑	(Li et al., 2018b)
	BBR	SH-SY5Y cells, high glucose	0.1–10 nM, 24 h	ROS production, nucleus condensation, and apoptosis↓; neurite outgrowth↑	Bcl-2 expression↑; cytochrome c release↓; Nrf2 and HO-1↑; ROS production↓; Nrf2 siRNA abolished BBR-induced HO-1, NGF, neurite outgrowth and ROS decrease	(Hsu et al., 2013)
	BBR	Wistar rats, STZ	25–100 mg/kg/day, 30 days	Cognitive performance↑; hyperglycemia and oxidative stress↓; MDA levels↓; GSH levels↑	Choline esterase (ChE) activity in the cerebral cortex and hippocampus↓	(Bhutada et al., 2011)
	BBR	SD rat, STZ + HFD; cerebral vascular smooth cells	50, 100, and 200 mg/kg/day, 8 weeks	Glucose levels↓; Ca(2+)channel (CaL) current densities↓; resting intracellular Ca(2+) ([Ca(2+)]i) level↓	α1C-subunit expressions of CaL↓; Ca(2+) releases from RyRs↓	(Ma et al., 2016b)
**Diabetic osteopathy**	BBR	SD rat, STZ+ HFD	100 mg/kg/day, 12 weeks	Reabsorption and irregular histomorphometry↓; bone mineral density↑	Serum osteocalcin and alkaline phosphatase (ALP) activity↑; TRAP↓; urinary 8-OHdG (DNA damage)↓; activities of SOD, catalase, glutathione peroxidase and GST↑	(Xie et al., 2018)
	BBR	Wistar albino rats, STZ+nicotinamide	100 mg/kg/day, 12 weeks	Blood glucose↓; HbA1c levels↓; urinary calcium↓; bone histomorphometry↑	Serum TRAP, mRNA expression of RANKL↓; PPAR-γ, Runx2, OPG, osteocalcin, and AMPK levels↑	(Adil et al., 2017)
**Diabetic enteropathy**	BBR	Wistar rats, STZ + HFD	93.75, 187.5, and 375 mg/kg/day, 10 weeks	Fasting and postprandial blood glucose↓; insulin resistance index and TG↓; immune cells in mesenteric lymph nodes↑; intestinal barrier damage↓	Intestinal TLR4, MyD88, and phosphorylation of IKKβ expressions↓; LBP and CD14 mRNA levels↓; IL-1β, MIF, and TNF-α mRNA ↓; IL-4 and IL-10 mRNA ↑ in intestinal tissue↑; secretion of GLP-1↑; production of GIP, amylin↑ and ghrelin↓ at high dose; OCLN, ZO-1 expressions↑; GFAP upregulation↓	(Gong et al., 2017)
	BBR	SD rat, STZ+ HFD	100 mg/kg, 2 weeks	Restoration of intestinal villi/mucosa structure; infiltration of inflammatory cells↓; plasma LPS level↓	Levels of ZO1 and GLP2↑	(Shan et al., 2013)
**Diabetic retinopathy**	BBR	Human retinal Müller cells+ native-LDL or HOG-LDL	0–20 µM, 1 h	HOG-LDL-induced Müller cell injury↓; autophagy and apoptosis↓	ATG-5 and beclin-1↓; ratio of LC3II/LC3I↓; cleaved PARR and cleaved caspase3↓; ROS production↓; expression of NOX4↓; expressions of Nrf2 and GPX-1↑; VEGF/PEDF ratio↓; protein levels of iNOS and ICAM-1↓; mRNA expressions of IL-6, IL-8, and TNF-α↓; AMPK expression↑	(Fu et al., 2016)
	BBR	Human retinal endothelial cells+ leukocytes freshly of non-diabetic or diabetic patients	0–50 µM, 24 h	Leukocytes of diabetic patients mediated killing of retinal endothelial cells *in vitro*↓	ICAM-1 (on endothelial cells) and CD18 (on leukocytes)↑; activation of NF-κB↓ and antioxidant enzymes (SOD, CAT, and GSH-Px)↑ in the retina	(Tian et al., 2013)
	Brb	Human retinal pigment epithelial cells, IL-1β or TNF-α	1–25 µM, 30 min		IL-8 and MCP-1 expression↓; NF-κB translocation↓	(Cui et al., 2006)

↓ indicates inhibition/reduction while ↑ indicates increase/promotion; BBR, berberine; Cop, coptisine; Pal, palmatine; Jat, jatrorrhizine; Brb, berberrubine; Col, columbamine.

## Anti-Diabetes Activities of *Rhizoma Coptidis*

### Adjustment of Glycometabolism

#### Inhibition of Digestion and Absorption of Sugar in the Digestive Tract

α-Glucosidase and disaccharidases are necessary for conversion of carbohydrates into simple sugars and their absorption in the intestine (Liu et al., 2010a; Hui et al., 2017). RC, coptisine, epiberberine, jatrorrhizine, berberine, and palmatine possess strong α-glucosidase inhibitory effects (Li et al., 2012b; Ge et al., 2014; Zhou et al., 2014; Chen et al., 2017a). It has been reported that berberine significantly inhibited the activity of disaccharidases in protein kinase A-dependent pathway and reduced the mRNA expressions of sucrase-isomaltase complex in diabetic rats and normal rats (Liu et al., 2010a). Berberrubine dose-dependently decreased the activity of intestinal α-glucosidase, and interestingly, berberrubine (50 mg/kg) showed stronger hypoglycemic effect than berberine (120 mg/kg) (Yang et al., 2017b). Therefore, by inhibition of carbohydrate digestion, RC directly inhibits increases in postprandial blood glucose levels.

#### Promotion of Glucose Uptake and Catabolism

Insulin (INS) induces the activation of insulin receptors, which leads to the recruitment of insulin receptor substrate (IRS) protein and activation of PI3K/Akt/AMPK, as well as glucose transporters (GLUTs) which promote glucose uptake (Wilcox, 2005; Hu et al., 2017). Studies have shown that RC polysaccharides decreased fasting blood glucose (FBG) by regulating JNK/IRS1/PI3K pathway (Jiang et al., 2015a), improved the phosphorylation of AMPK in muscle and liver, and mediated glucose uptake in 3T3-L1 adipocytes by regulating PI3K/AMPK pathway (Cui et al., 2016). Berberine increased mRNA and protein expressions of insulin receptor in various human cell lines (Zhang et al., 2010). It promoted the expression of GLUT-4 mRNA in skeletal muscle (Liu et al., 2015) and improved the expression of insulin receptor substrate (InsR) 1, GLUT-1, and GLUT-3 in brain (Sandeep and Nandini, 2017). Palmatine also promoted glucose uptake of L6 cells by promoting the expression of GLUT-4 (Sangeetha et al., 2013). It has been demonstrated that the natural mass ratio of berberine and ferulic acid was approximately 20:1, and their synergistic effect indicated that RC has better clinical application value than berberine (Chen et al., 2012). Dietary polyphenols are effective supplements for the treatment of T2DM (Cao et al., 2018a). Octadecyl caffeate, a dietary polyphenol of RC, stimulated glucose uptake at a dose of 50 μg/ml in skeletal muscles (Yang et al., 2014a). Coptisonine from RC also stimulated glucose uptake at a dose of 25 μg/ml in skeletal muscles (Yang et al., 2014a).

When cells are in a state of low energy charge, AMPK is activated to promote glucose uptake and utilization (Merrill et al., 1997). In HepG2 hepatocytes and C2C12 myotubes, berberine bi-directionally regulated the expression of AMPK and promoted glucose metabolism by stimulating glycolysis, an effect which may not be related to AMPK activity (Xiao et al., 2018). Similarly, a study has also indicated that AMPK plays an important but not dependent role in the ability of berberine to promote glucose uptake (Xu et al., 2014).

#### Promotion of Glycogen Synthesis and Inhibition of Gluconeogenesis

Glucokinase, a rate-controlling enzyme in glycogen synthesis in the liver, and an important regulator of diabetes, catalyzes the glucose to glucose-6-phosphate (Teng et al.; Matschinsky et al., 2011). In a study, berberine promoted glucokinase activity and up-regulated the expression of hepatocyte nuclear factor 4 alpha (HNF-4α) in mouse primary hepatocytes (Yan et al., 2008). Berbamine (100 mg/kg) also improved glycogen content in insulin-dependent tissues (Sankaranarayanan et al., 2018). Xia et al. (2011) have shown that berberine improved FBG by directly inhibiting gluconeogenesis in insulin-independent way in liver. In different studies, berberine inhibited gluconeogenesis by 1) regulating LKB1-AMPK-TORC2 pathway (Jiang et al., 2015b), 2) mediating expressions of HNF-4α and its downstream miR122 (Wei et al., 2016), 3) inhibiting sirtuin (SIRT)-3 expression to block the release of glucagon and the downstream targets of gluconeogenesis (Zhang et al., 2018), 4) inhibiting the key gluconeogenesis enzyme expression, such as phosphoenolpyruvate carboxykinase (PEPCK) and glucose-6-phosphatase (G6Pase) (Xia et al., 2011; Jiang et al., 2015b).

#### Protection of Islet **β** Cells

Cytokines produced by the infiltration of immune cells in islets are important mediators of β cell destruction in T2DM. RC extract and berberine exerted protective effect on cytokine-induced apoptosis of β cells (Kim et al., 2007; Wang, 2013). When Rin-5F cells were treated with RC and berberine, glucose-stimulated insulin release, cell cycle, lipotoxic islet cell proliferation, and protein expression of poly(ADP-ribose) polymerase 1 (PARP-1) were altered and improved, which suggest that RC may protect β cells by improving islet β cell proliferation and PARP-1 protein expression (Jiang et al., 2017). RC extract inhibited cell apoptosis and necrosis of Rin-m5F cells induced by S-nitroso-N-acetylpenicillamine *via* elimination of disruption of mitochondrial membrane potential (Kwon et al., 2005). In addition, islet cells pretreated with RC extract retained insulin secretion even after treatment with interleukin (IL)-1β (Kwon et al., 2005). Toll-like receptor 4 (TLR4) inflammatory pathway mediates β cell injury in T2DM. Berberine attenuated lipopolysaccharide (LPS)-induced inflammatory and apoptotic responses in β cells *via* TLR4-independent JNK/NF-κB pathway (Wang, 2013). Berberine also inhibited apoptosis of β cells by down-regulation of Bax/Bcl-2 gene expressions (Chueh and Lin, 2012). RC polysaccharides also significantly inhibited the formation of protein glycation and advanced glycation end products (AGEs) in pancreas and suppressed hyperglycemia-induced damage to the pancreas (Yang et al., 2016b).

Oxidative stress is a pathological process of tissue and organ damage caused by imbalance between oxidants and antioxidants *in vivo*, which is a crucial risk factor for diabetes mellitus (Giacco and Brownlee, 2010; Rochette et al., 2014). Silent information regulator 1 (SIRT1) exerts both anti-oxidative and anti-inflammatory effects (Chen et al., 2013b). The SIRT1 is the target gene of miR-106b, and over-expression of miR-106b can reverse the anti-oxidative effect of berberine in NIT-1 cells cultured in a high-glucose medium (Chen and Yang, 2017). This suggests that berberine protects islet β cells partially *via* SIRT1/miR-106b pathway.

#### Promotion of Insulin Secretion

Oral administration of berbamine (50,100,200 mg/kg) for 56 days improved insulin secretion in a dose-dependent manner in T2DM rats (Sankaranarayanan et al., 2018). Oral administration of palmatine, jatrorrhizine, or magnoflorine (10, 20, and 40 mg/kg) raised the serum insulin level in glucose-fed rats (Patel and Mishra, 2011). RC polysaccharides also restored islet size and increased insulin secretion in a T2DM model (Cui et al., 2016).

Incretin is a gut-derived hormone, which responses to nutrient intake (mainly glucose and fat). It is considered to be the strongest insulin-secreting hormone, and it inhibits appetite and glucagon secretion and delays gastric emptying (Yabe and Seino, 2011). Glucagon-like peptide-1 (GLP-1) is an intestinal hormone with incretin-like function (Chu et al., 2007). It is rapidly inactivated by dipeptidyl peptidase 4 (DPP-4) enzyme (Simona and Itamar, 2011). Thus, inhibition of DPP-4 and increase in GLP-1 level *in vivo* can improve the responsiveness of β cells to glucose and promote insulin secretion, while inhibiting glucagon secretion, thereby reducing FBG and postprandial blood glucose (Drucker and Nauck, 2006; Del Prato et al., 2011). Berberine is a DPP-4 inhibitor (Al-Badri et al., 2018). In a study, it was shown that berberine reduced plasma glucose level of streptozotocin (STZ)-induced diabetic rats by inhibiting DPP-4 and increasing GLP-1 level in intestine (Wang et al., 2016a). Berberine is a ligand for bitterness receptors. Studies have shown that berberine stimulates GLP-1 secretion by activating intestinal bitterness receptor TAS2R38 in a phospholipase C (PLC)-dependent manner (Yu et al., 2015). The L-cells are located in the colon and are responsible for secretion of GLP-1. Berberine also improved GLP-1 expression by up-regulating the expression of GPR43 (a receptor of SCFAs) and suppressed ATP overproduction-induced mitochondrial stress in L-cells (Sun et al.). After GLP-1 activation, adenylate cyclase is activated and cyclic adenosine monophosphate is produced, resulting in activation of the second messenger pathway and closure of the adenosine triphosphate-dependent potassium channel. Increased intracellular potassium induces depolarization and leads to calcium influx through voltage-dependent calcium channels. The increase in intracellular calcium stimulates the migration and exocytosis of insulin (Cicero and Tartagni, 2012). Uncoupling protein 2 (UCP2) regulates glucose-stimulated insulin secretion. A study has shown that berberine inhibited oxidative stress and restored insulin secretion in high-glucose-treated INS-IE cells and db/db mice islets *via* the AMPK/UCP2 cascade (Liu et al., 2014).

#### Mitigation of Insulin Resistance

The development of T2DM is usually associated with insulin resistance and impaired glucose uptake in peripheral tissues (DeFronzo, 2004). Peroxisome proliferator-activated receptor (PPAR) γ is a well-known anti-diabetic insulin sensitizer receptor related to fat production and glucose homeostasis (Sharma and Staels, 2007; Li et al., 2009; Cheatham, 2010; Sangeetha et al., 2013). Five known alkaloids, berberine, epiberberine, coptisine, palmatine, and magnoflorine inhibited the downregulation of PPARγ in 3T3-L1 cells in a dose-dependent manner (Choi et al., 2014). In L6 cells, palmatine positively modulated the expressions of PPARα and PPARγ (Sangeetha et al., 2013). Berberine also reduced fasting serum insulin and enhanced glucose uptake and insulin sensitivity through several mechanisms including 1) up-regulation of the expression of insulin receptor in a protein kinase C (PKC)-dependent manner (Kong et al., 2009); 2) down-regulation of the expressions of resistin and leptin in insulin resistant 3T3-L1 adipocytes (Tu et al., 2016); 3) regulation of bone morphogenetic protein (BMP) 4 transcriptional pathways and brown adipose tissue transcriptional pathways in white/brown adipose tissues (Li et al., 2016); and 4) regulation of transcription of sterol regulatory element-binding transcription factors (SREBPs), liver X receptors (LXRs), and PPARs in liver and visceral white adipose tissue (Liu et al., 2010b; Li et al., 2011).

In some insulin-resistant patients, protein tyrosine phosphatase 1B (PTP1B) interacted with insulin receptor and induced insulin receptor dephosphorylation, resulting in inactivation of insulin signaling pathway (Ahmad et al., 1997; Combs, 2010). Alkaloids of RC, such as berberine, epiberberine, magnoflorine, and coptisine showed inhibitory effects on PTP1B due to their higher affinity and tighter binding capacity to the active site of PTP1B (Bustanji et al., 2006; Choi et al., 2015). Berberine and epiberberine showed mixed-type inhibition against PTP1B, while magnoflorine and coptisine noncompetitively inhibited PTP1B (Choi et al., 2015). Retinol-binding protein 4 (RBP4), a cytokine secreted by adipocytes, led to insulin resistance and negatively correlated with the expression of GLUT-4 in insulin-resistant states (Yang et al., 2005; Graham et al., 2006). Berberine lowered serum RBP4 level and up-regulated the expression of GLUT-4, thereby promoting glucose uptake in insulin-resistant cells (Zhang et al., 2008).

#### Regulation of Gut Microbiota

T2DM is associated with imbalance in gut microbiota (Cani et al., 2008). Regulation of gut microbiota improved glucose intolerance and changed inflammation and metabolic status (Han et al., 2011). After administration of RC extract, the diversity of gut microbiota in T2DM rats was changed, suggesting that RC ameliorated the disordered gut microbiota in T2DM (Ning-Ning et al., 2017). Due to the poor absorption of berberine, the regulation of intestinal microorganisms has been assumed to be one of the mechanisms involved in its anti-diabetic effects (Han et al., 2011). The balance between beneficial gut bacteria (e.g., SCFAs-producing bacteria) and opportunistic pathogens (e.g., endotoxin-producing bacteria and sulfate-reducing bacteria) is important for intestinal homeostasis (Qin et al., 2012). Berberine inhibited high-fat diet (HFD)–induced reduction of intestinal butyric acid bacteria, i.e., *Blautia, Bacteriodes, Butyricoccus, Allobaculumand*, and *Phascolarctobacterium*, resulting in anti-inflammatory properties and improved insulin resistance (Jakobsdottir et al., 2013; Zhang et al., 2015; Wang et al., 2017). It has been reported that dihydroberberine has better hypoglycemic effect than berberine because it is more easily absorbed by intestinal epithelial cells (Turner et al., 2008). Further studies have shown that dihydroberberine is quickly converted to berberine after absorption, suggesting that berberine is the real hypoglycemic agent. Moreover, gut microbiota can convert berberine into dihydroberberine, thereby enhancing the absorption of berberine (Feng et al., 2015).

### Anti-Diabetic Complications Effects of RC

#### Diabetic Vascular Dysfunction

Endothelial dysfunction, as characterized by impaired endothelium-dependent vasodilation and reduced bioavailability of nitric oxide (NO), is closely related to T2DM (Huang, 2003; Félétou and Vanhoutte, 2006; Forstermann and Munzel, 2006). Studies have shown that berberine promoted insulin-induced vasodilatation in rats and ameliorated cell viability and autophagy in human artery endothelial cells by activation of the InsR/AMPK/Akt/eNOS pathway (Geng et al., 2016). In aortic endothelial cells, berberine prevented hyperglycemia-induced endothelial injury and enhanced vasodilatation *via* down-regulation of endothelial NO and reactive oxygen species (ROS) synthase through AMPK-dependent approaches (Wang et al., 2009b). In palmitic acid-treated human umbilical vein endothelial cells, berberine also significantly increased the bioavailability of NO and reduced the production of ROS with the up-regulation of p-AMPK protein levels (Zhang et al., 2013). Altogether, these findings suggest that the activation of AMPK is necessary for berberine to regulate endothelium-dependent vasodilation. In rat brain, berberine protected microvascular endothelial cells from hypoxia/high-glucose-induced impairment through the SIRT1/HIF-1α/VEGF pathway (Mi et al., 2019).

#### Diabetic Heart Disease

Diabetic cardiomyopathy is one of the most common complications of T2DM with cardiomyocyte hypertrophy, ventricular enlargement, cell apoptosis, and myocardial interstitial fibrosis as the main pathological changes (Campbell et al., 2011; Bulani et al., 2019; Feidantsis et al., 2018). These complications lead to heart failure (Waldman et al., 2018). Berberine inhibited high-glucose-induced cardiomyocyte hypertrophy by activating the PPARα/NO signaling pathway (Wang et al., 2013b). Berberine improved cardiac fibrosis in diabetic rats through 1) down-regulation of the expressions of myocardial insulin-like growth factor 1 (IGF-1) receptor–regulated matrix metalloproteinases (MMP)-2/MMP-9 and 2) inhibition of expressions of transforming growth factor-β1 (TGF-β1) and connective tissue growth factor (CTGF), thereby reducing α-smooth muscle actin (α-SMA) and collagen 1 and collagen 3 levels (Lu et al., 2016; Li et al., 2018a).

The tolerance of diabetic patients to myocardial ischemia is significantly reduced by microvascular damage, leading to increase in myocardial infarction area and poor recovery of cardiac function after reperfusion (Iwakura et al., 2003; Kurisu et al., 2003). Berberine exerted protective effects against ischemic arrhythmias in T2DM rats possibly by regulation of ion channels and recovery of the balance of electric parameters (Wang et al., 2011; Wang et al., 2012). It also inhibited apoptosis and improved cardiac function after ischemia/reperfusion by activating AMPK and PI3K/Akt/GSK3β/eNOS signaling pathway in diabetic rats (Chen et al., 2014; Chang et al., 2016).

#### Diabetic Hyperlipidemia

Abnormalities in lipid metabolism, including disturbance of fatty acids and lipoproteins, are among the major factors contributing to an increased cardiovascular risk in T2DM (Verges, 2015; Soran et al., 2016). Diabetic hyperlipidemia is usually reflected in increase in free fatty acids (FFA), triglyceride (TG), total cholesterol (TC), low-density lipoprotein cholesterol (LDL-C), apolipoprotein B (ApoB), and decreased levels of high-density lipoprotein cholesterol (HDL-C) (Betteridge, 1999; Goldberg, 2001). In a T2DM model, RC reduced blood lipid level and down-regulated expressions of sterol regulatory element-binding protein 1c (SREBP-1c) and SREBP cleavage-activating protein (SCAP) in the liver (Liu et al., 2018). Berberine mitigated disorder in lipid metabolism partly by decreasing expressions of TNF-α and FFA, while increasing the activity of lipoprotein lipase (Zhou and Zhou, 2010). Jatrorrhizine also down-regulated SREBP-1c and fatty acid synthase (FAS) mRNA levels in liver, while up-regulating the expressions of PPAR-α and carnitine pecalmitoyltransferase 1A (CPT1A), thereby lowering serum TG, TC, and LDL-C levels and increasing serum HDL-C level (Yang et al., 2016a). By up-regulation of HNF-4α and fetoprotein transcription factor (FTF), columbamine promoted cholesterol 7α-hydroxylase (CYP7A1) promoter activity and enhanced the conversion of cholesterol to bile acid and its excretion in the feces (Wang et al., 2016b). Berberrubine exerted lipid-lowering effects through regulation of low-density lipoprotein receptor (LDLR) and pro-protein convertase subtilisin/kexin type 9 (PCSK9) in the extracellular signal-regulated kinase 1/2(ERK1/2) signaling pathway (Cao et al., 2018b). RC polysaccharides also reduced the levels of TG and TC in diabetic mice (Jiang et al., 2013).

#### Diabetic Nephropathy

Hyperglycemia leads to abnormal glycosylation of proteins (Lassila et al., 2004), disorders in lipid metabolism (Sieber et al., 2010), abnormal changes in glomerular hemodynamics (Reudelhuber, 2010), and activation of protein kinase pathways (Soetikno et al., 2011). These changes eventually lead to local renal microcirculation disorders, thickening of glomerular basement membrane (GBM), deposition of extracellular matrix (ECM), glomerulosclerosis, and interstitial fibrosis. Berberine inhibits or delays the occurrence and development of diabetic nephropathy through a variety of mechanisms, as shown in **Table 1** and **Figure 4**.

Berberine significantly reduced the kidney weight, urinary protein content, and blood urea nitrogen (BUN) levels in T2DM (Liu et al., 2008a). The TGF-β1 plays an extremely important role in the therapeutic effect of berberine. Through regulation of upstream targets such as nuclear factor-erythroid 2–related factor 2 (Nrf2) (Zhang et al., 2016), AGEs (Qiu et al., 2017), and activator protein-1 (AP-1) (Lan et al., 2014), berberine inhibited the expression of TGF-β1, thereby influencing downstream signals *via* 1) activation of PI3K/Akt pathway (Wang et al., 2018c), 2) inhibition of the expressions of Smad2 and Smad3, and 3) suppression of the matrix metalloproteinases (MMPs)/tissue inhibitor of matrix metalloproteinases (TIMPs) system (Ni et al., 2015). These changes ultimately lead suppression of the thickening of GBM, deposition of ECM, glomerulosclerosis, and interstitial fibrosis.

The suppression of p38MAPK up-regulates the expressions of cAMP-response element binding protein (CREB) and fibronectin (FN), suggesting that p38MAPK participates in the accumulation of ECM. Berberine partly reduced high-glucose-induced FN expression and collagen synthesis in mesangial cells by suppression of p38MAPK (Liu et al., 2009). It inhibited glomerular mesangial cell proliferation through regulation of PGE2-EPs-G protein-adenylyl cyclase (AC)-cAMP-Ca^2+^ signaling pathway (Tang et al., 2013; Wang et al., 2013a; Yang et al., 2014b; Ni et al., 2016) and inhibited Snail expression through regulation of Notch signaling pathway, leading to suppression of the high-glucose-induced epithelial-to-mesenchymal transition (Yang et al., 2017a). It has been reported that the anti-inflammatory and anti-oxidative activities of berberine partly contributed to its therapeutic effects on diabetic nephropathy, including inhibition of the expressions of Nrf2/HO-1, NF-κB, and the production of ROS (Zhang et al., 2016; Zhu et al., 2018).

Cellular polyol pathway is activated by persistent high-glucose stimulation that leads to cellular dysfunction accompanied by oxidative stress injury (Sytze et al., 2013). The alkaloids berberine, palmatine, coptisine, and jatrorrhizine, which contain isoquinoline/bis(isoquinoline) and related ring structures, have exhibited strong aldose reductase (AR) inhibitory activities and can be used to control diabetic complications including nephropathy (Liu et al., 2008a; Liu et al., 2008b; Gupta et al., 2014).

#### Diabetic Encephalopathy

Chronic and persistent hyperglycemia may impair central nervous system function, affect cognitive function, and lead to dementia (Luchsinger et al., 2001). As shown in **Table 1** and **Figure 5**, RC exerts therapeutic effect on diabetic encephalopathy through different channels. Total alkaloids of RC alleviated cognitive impairment in type 2 diabetic rats by reducing Aβ deposition and enhancing insulin signaling (Li et al., 2018c). Palmatine inhibited the expression of P2X receptor in hippocampus, resulting in attenuation of diabetic neuropathy, which may be related to the inhibition of ERK1/2 phosphorylation and the release of tumor necrosis factor (TNF)-α and IL-1β in hippocampus (Shen et al., 2018). Berberine easily penetrates the blood–brain barrier, and its elimination is slow in brain (Wang et al., 2005). It affects diabetic encephalopathy through the following ways: 1) regulation of SIRT1/endoplasmic reticulum (ER) stress pathway and Nrf2/HO-1 pathway (Hsu et al., 2013), leading to enhancement of the antioxidant capacity of nerve cells (Li et al., 2018b); 2) restoration of hippocampal synaptic plasticity (Kalalian-Moghaddam et al., 2013) and activation of PI3K/Akt/GSK-3β/tau pathway to prevent axonopathy (Chen et al., 2017b); 3) reduction of inflammation by inhibition of NF-κB/MAPK pathway (Zhou et al., 2016; Chen et al., 2017b); 4) inhibition of the expression of beta-site amyloid precursor protein-cleaving enzyme 1 (BACE-1) leading to reduction of Aβ deposition (Chen et al., 2017b); 5) inhibition of neuronal apoptosis by regulation of mitochondrial pathway (Friedemann et al., 2014); 6) prevention of pain through inhibition of the PKC/TRPV1 pathway; and 7) promotion of the expression of GLUT3 to enhance glucose absorption and utilization in the brain.

Diabetic vascular dysfunction is usually accompanied by impairment of intracellular Ca^2+^ handling in vascular smooth muscle cells (VSMCs) (Fernández-Velasco et al., 2014). Hyperglycemia induces increases in intracellular Ca^2+^ by activating long-lasting voltage-dependent Ca^2+^ channels, thereby promoting Ca^2+^ influx and altering Ca^2+^ release from the ryanodine receptors (RyRs) in VSMCs (Navedo et al., 2010; Krishnamoorthynatarajan and Koide, 2016). In cerebral VSMCs, berberine regulated intracellular Ca^2+^ handling by inhibition of current density and α1C subunit expression in long-lasting voltage-dependent Ca^2+^ channels, reducing the release of RyRs and the concentration of intracellular Ca^2+^, leading to reduction in blood pressure and reversal of impaired vascular relaxation in STZ-induced T2DM model (Ma et al., 2016b; Ma et al., 2017).

#### Diabetic Osteoporosis

Diabetic osteoporosis is a serious complication of diabetes mellitus in the skeletal system, which is characterized by destruction of bone tissue structure, reduction in bone mass, increase in bone fragility, and susceptibility to fractures (Kanazawa, 2017; Rathinavelu et al., 2018; Zhang et al., 2019a). Hyperglycemia also stimulates oxidative stress in bone tissue and promotes bone reabsorption of osteoclasts (Cunha et al., 2014). In STZ and HFD-induced diabetic osteoporosis, berberine significantly up-regulated the activity of serum antioxidants, thereby lowering oxidative stress-induced damage to DNA (Xie et al., 2018). Hyperosmotic state induced by hyperglycemia affects osteoblast formation by promoting urinary calcium excretion and reducing blood calcium concentration and bone calcium deposition (Moerman et al., 2004). Insulin promotes the metabolism of osteoblasts and increases bone formation, whereas the reduction of insulin secretion may result in decreased metabolism and bone formation by osteoblasts (Hamann et al., 2012). Bone marrow mesenchymal stem cell differentiation into adipocytes is promoted by PPAR-γ, which also inhibits osteoblast differentiation, thereby reducing bone mineral density and increasing fracture risk (Kahn et al., 2006; Smith et al., 2015). Berberine treatment alone, or in combination with pioglitazone, remarkably ameliorated expressions of AMPK and PPAR-γ mRNA and reversed abnormal urinary calcium and serum insulin levels in diabetic rats (Adil et al., 2017). In some studies on STZ-induced diabetic rats, bone resorption markers such as urinary calcium and serum tartrate-resistance acid phosphatase (TRAP) were activated but were down-regulated by berberine (Adil et al., 2017; Xie et al., 2018). Receptor activator for nuclear factor-κB ligand (RANKL) is a binding partner for osteoprotegerin (OPG). Berberine improved osteoclast and osteoblast differentiation by regulating RANKL and OPG expressions in bone marrow stromal cells (Xue et al., 2012). Runt-related transcription factor 2 (Runx2) regulates the expressions of various osteoblast-related ECM proteins in osteoblasts, including osteocalcin (a bone formation marker) to promote bone formation (Haxaire et al., 2016; Liu et al., 2016). Berberine treatment alone or in combination with pioglitazone remarkably ameliorated RANKL, OPG, Runx2, and osteocalcin in diabetic rats (Adil et al., 2017).

#### Diabetic Enteropathy

Compared with non-diabetic patients, diabetic patients are more likely to have gastrointestinal symptoms, including abdominal pain, abdominal discomfort, heartburn, and diarrhea (Bytzer et al., 2001; Bytzer et al., 2002). Diabetes mellitus is accompanied by inflammation and oxidative stress, which lead to impaired intestinal permeability, and transfer of enteric LPS into the blood, eventually leading to endotoxemia (Cani et al., 2009; Yamamoto and Yamamoto, 2013). In STZ plus HFD-induced diabetic model, berberine regulated TLR4/MyD88/NF-κB signaling pathway in intestinal tissues (Gong et al., 2017) and promoted the expressions of glutamine-induced glucagon-like peptide-2 (GLP2) and tight junction protein zonula occludens 1 (ZO1), resulting in significant recovery of intestinal villus/mucosa structure and less infiltration of inflammatory cells, amelioration of intestinal barrier abnormalities, and endotoxemia (Shan et al., 2013). The regulation of intestinal flora disorders has already been described in 1.7.

#### Diabetic Retinopathy

Diabetic retinopathy is the most common microvascular complication in diabetic patients (Calderon et al., 2017). Hyperglycemia induces blood-retinal barrier injury. Low-density lipoprotein (LDL) leaks through blood-retinal barriers and is subsequently modified by oxidation and glycosylation to products which induced death in several types of human retinal cells (Fu et al., 2012; Fu et al., 2014). Müller cells are the major glia of the retina that provide support to retinal neurons. They control blood flow, modulate neuronal activity and glucose metabolism, and maintain the blood-retinal barriers (Canning et al., 2007; Ahmad et al., 2011). Berberine inhibited highly oxidized glycated-LDL-induced damage to Müller cells by activating the AMPK pathway (Fu et al., 2016). Studies have shown that leukocytes from diabetic mice caused direct injury and death of retinal endothelial cells (Li et al., 2012a; Talahalli et al., 2013). The secretion of inflammatory mediators leads to the recruitment and exudation of leukocytes, thereby enhancing the inflammatory processes (Chen et al., 2018). Berberine significantly inhibited leukocyte adhesion to human retinal endothelial cells as well as leukocyte-mediated killing of endothelial cells by inhibiting the downregulation of intercellular cell adhesion molecule-1 (ICAM-1) (on endothelial cells) and integrin ligand integrin beta-2 (CD18) (on leukocytes), suppressing the activation of NF-κB and improving antioxidant enzymes (SOD, CAT, and GSH-Px) in the retina (Tian et al., 2013). In IL-1β or TNF-α evoked human retinal pigment epithelial cell line (ARPE-19), berberrubine dose-dependently inhibited the expression and protein secretion of IL-8 and monocyte chemotactic protein-1 (MCP-1) through regulation of NF-κB pathway (Cui et al., 2006). AR is an important enzyme in the polyol pathway, which plays a key role in the occurrence of diabetic complications (Jesús Angel et al., 2003). Studies have shown that RC extract and its alkaloids (epiberberine, coptisine, groenlandicine, jatrorrhizine, palmatine, and magnoflorine) exhibited moderate inhibitory activities on rat lens aldose reductase (RLAR), which partly contributed to the anti-diabetic retinopathy effect of RC (Jung et al., 2008; Patel and Mishra, 2012).

## Pharmacokinetic Properties of Rhizome Coptidis in T2DM

It is generally known that patients are the consumers of drugs. Therefore, studies on the pharmacokinetic properties of RC in pathological conditions of T2DM are quite necessary. Pathological conditions of T2DM, including hyperglycemia, hyperlipidemia, and autonomic neuropathy, affect hemorheological parameters and change the absorption and distribution of drugs (Stacher et al., 2003). It has been reported that T2DM delayed gastric emptying, resulting in prolonged transit time of drugs in gastrointestinal tract, thereby promoting their absorption (Chiu et al., 2014). Moreover, it has been found that pharmacokinetic properties of berberine in normal and T2DM rats were quite different, and that the bioavailability of berberine was improved in T2DM rats (Jia et al., 2017).

Relative to normal rats, T2DM rats exhibited increased pharmacokinetic parameters for berberine (20 mg/kg). Cmax, t_1/2_, and AUC (17.35 ± 3.24 *vs* 34.41 ± 4.25 μg/L, 3.95 ± 1.27 *vs* 9.29 ± 2.75 h, 151.21 ± 23.96 *vs* 283.81 ± 53.92 μg⋅h/L, respectively) and oral clearance rates (134.73 ± 32.15 *vs* 62.55 ± 16.34 L/h/kg) were decreased significantly. Intestinal ATP-dependent efflux pump P-glycoprotein (PGP) is distributed widely in the intestinal epithelium and hepatocytes, and it reduces drug efficiency of protoberberine alkaloid (Pan et al., 2010; Zhang et al., 2019b). A study revealed that intestinal P-GP was impaired in STZ-induced T2DM model, which partly enhanced the absorption of the five protoberberine alkaloids (berberine, palmatine, coptisine, epiberberine, and jatrorrhizine) (Yu et al., 2010). Small structural differences (e.g., the location and number of hydroxyl groups) led to different binding affinities to P-GP (e.g., berberine and coptisine) (Cui et al., 2015; Zhang et al., 2019b). In normal rats, berberine was quickly distributed in organs, and the concentrations in organs were higher than that in blood (Tan et al., 2013). Similarly, in HFD and STZ-induced diabetic rats, berberine was quickly distributed in the liver, kidney, and lung, and the concentrations in organs were higher than that in blood (Cui et al., 2018).

Berberine is the main active component of RC. However, the bioavailability of berberine is very low. Nevertheless, clinical applications of RC showed that it has high hypoglycemic effects. RC is generally used in the form of formula in traditional Chinese medicine, such as *Jiao-Tai-Wan* and *San-Huang-Xie-Xin-Tang*. It has been revealed that the Cmax and AUC(0–∞) of *Jiao-Tai-Wan* were higher than those of RC in diabetic mice, and that cinnamon in *Jiao-Tai-Wan* promoted bioavailability of RC (Chen et al., 2013a). In STZ and HFD-induced T2DM model, the pharmacokinetic curves (especially AUC, Tmax, and Cmax) of coptisine, berberine, and palmatine of *San-Huang-Xie-Xin-Tang* in normal and T2DM rats were quite different (Wei et al., 2017).

## Structure–Activity Relationships of Protoberberine Alkaloids in T2DM

Substitutions at positions C-2, C-3, C-9, and C-10 play important roles in anti-diabetic effects of protoberberine alkaloids of RC. It has been shown that the binding affinity to β-cell sulfonylurea receptors disappeared when the methylenedioxy group in C-2 or C-3 was replaced by other groups (Bian et al., 2006). When C-9 and C-10 were both substituted by methoxyl group, the hypoglycemic effect was stronger. However, when C-9 or C-10 was substituted by only one methoxy or benzyloxy group, the hypoglycemic effect was reduced (Bian et al., 2006). Moreover, it has been demonstrated that berberine and coptisine had more significant hypoglycemic effects than palmatine, epiberberine, and jatrorrhizine (Ma et al., 2016a). Berberine had the highest hypoglycemic effect partly due to its methoxyl groups in C-9 and C-10 positions (Ma et al., 2016a). This is consistent with the results obtained in another study (Chen et al., 2012). Therefore, methylenedioxy groups in C-2, C-3 and two methoxy substituents in C-9, C-10 contribute greatly to the hypoglycemic effect of protoberberine alkaloids of RC. The methylenedioxy group in D-ring may contribute to the inhibitory activity of RLAR (Jung et al., 2008). When the quaternary salts were reduced to tertiary amines, the binding affinity of berberine to β-cell membranes disappeared, and the aromatic C ring became unnecessary for the hypoglycemic effects of berberine (Bian et al., 2006). The electrostatic effect of positively charged nitrogen atoms is important for anti-diabetic activity of alkaloids. A study has shown that the positively charged isoquinolinium nitrogen atom of berberine could bind to the anionic acid site ASP48 of PTP1B and competitively inhibit the recombination of PTP1B (Bustanji et al., 2006).

## Conclusion

Chinese herbal medicines with multiple-components are often used to successfully manage miscellaneous diseases, particularly chronic diseases triggered by multiple factors (Gao et al., 2017). RC has been shown to possess beneficial effects on various diseases by multi-targeted therapies (Wang et al., 2018a). In this review, the most relevant articles were evaluated to reveal how RC exerts curative effect on T2DM and its complications through multi-component and multi-target ways. In the present review, it has been shown that RC with multi-component and multi-target characteristics effectively treats T2DM and its complications by adjusting glycometabolism and inhibiting T2DM-induced damage to the kidneys, pancreatic islets, and other tissues and organs. However, the precise mechanism which underlies these effects has not yet been completely elucidated. The pharmacokinetic properties and structure-activity relationships of protoberberine alkaloids of RC in T2DM have been investigated. In protoberberine alkaloids, rather small structural changes can bring about significant differences in actions. Long-term clinical application has demonstrated that RC has good hypoglycemic effects, but bioavailabilities of protoberberine alkaloids (mainly active ingredients) are rather low, which cannot explain the clinical hypoglycemic effect. The problem may be partly explained in the following ways: 1) it is possible that the protoberberine alkaloids exert hypoglycemic effect by direct impact on the intestinal tract and intestinal flora, 2) the bioavailability of RC may be significantly improved in the pathological conditions of T2DM, 3) the synergistic effect between alkaloids, and between alkaloids and non-alkaloids (e.g., polysaccharides and ferulic acid), may have improved the hypoglycemic effect of RC. At present, the hypoglycemic effect of RC is mainly focused on the alkaloid fraction, while the effect of the non-alkaloid fraction is mostly neglected. It has been reported that after removing the six main protoberberine alkaloids, the rest still had hypoglycemic effects similar to that of berberine, especially at low concentrations (Chen et al., 2012).

With the increasing number of T2DM patients, it is of great significance to find safe and effective drugs for reducing blood glucose and preventing T2DM complications in natural medicines. Although RC has been applied in clinical treatment of diabetes for many years, there is still a big gap between theoretical research and clinical practice. More investigations, especially reliable clinical trials, including large-scale, rigorously controlled, and multicenter randomized controlled clinical trials, are needed to assess its long-term safety.

## Author Contributions

Q-WH is the corresponding author of the study. QR and JW are the first authors and responsible for collecting materials and writing the paper. LW, H-RZ and X-BY helped organizing the information and edited the article pictures. All authors read and approved the final manuscript.

## Funding

This work is financially supported by the project of administration of traditional Chinese medicine of Sichuan province (Code: 2018JC011) and by the National Chinese Medicine Standardization Project (Code: ZYBZH-Y-CQ-46).

## Conflict of Interest Statement

Author X-BY is employed by Xun Kang Pharmaceutical Co. Ltd, Ya’an, China.

The remaining authors declare that the research was conducted in the absence of any commercial or financial relationships that could be construed as a potential conflict of interest.

## Abbreviations

AMPK, AMP-dependent kinase; PI3K, phosphoinositol-3-kinase; Akt, protein kinase b; JNK, c-Jun N-terminal kinase; LKB1, liver kinase B1; TORC2, CREB-regulated transcription coactivator 2; NF-κB, nuclear factor kappa B; IκBα, NF-κB inhibitor alpha; IKKβ, inhibitor of NF-κB kinase subunit beta; VEGF, vascular endothelial growth factor; GSK3β, glycogen synthase kinase 3β; eNOS, endothelial nitric oxide synthase; HSP90, heat-shock protein 90; SphK, sphingosine kinases; S1P, sphingosine-1-phosphate; RhoA, Ras homolog gene family, member A; SCFAs, short-chain fatty acids; ROCK, Rho-associated protein kinase; RAGE, renal tumor antigen; cAMP, cyclic adenosine monophosphate; mTOR, mammalian target of rapamycin; MAPK, mitogen-activated protein kinase; NGF, nerve growth factor; MyD88, myeloid differentiation primary response gene 88; SOD, superoxide dismutase; CAT, catalase; GSH-Px, glutathione peroxidase; NOX4, NADPH oxidase 4; PGE2, prostaglandin E2; EPs, E-prostanoid receptors; HO-1, heme oxygenase 1; GRK, G protein-coupled receptor kinase; TRPV1, transient receptor potential cation channel subfamily V member 1; PSD95, postsynapticdensity 95; Aβ, β-amyloid; CHOP, C/EBP homologous protein; SphK1, sphingosine kinase 1; NQO1, NAD(P)H quinone dehydrogenase 1; GFAP, glial fibrillary acidic protein; ER stress, endoplasmic reticulum stress; eIF2α, eukaryotic initiation factor 2α; IRE-1**α**, inositol-requiring enzyme 1 alpha; PDI, protein disulfide isomerase; Sirt1, silent information regulator 1; SYN, synaptophysin; 8-OHdG, 8-hydroxydeoxyguanosine; CaL, Ca^2+^ channel; MIF, macrophage migration inhibitory factor; LBP, LPS-binding protein; GIP, gastric inhibitory polypeptide; OCLN, occludin-1; ATG-5, autophagy-related homologue 5; HOG-LDL, highly oxidized, glycated LDL; PEDF, pigment epithelium-derived factor; GPX-1, glutathione peroxidase 1; AUC, area under the concentration-time curve; Cmax, maximum plasma concentration; Tmax, time to Cmax; t1/2, reaction half-time; BMP4, bone morphogenetic protein 4.
